# Movement as Medicine for Cardiovascular Disease Prevention: Pilot Feasibility Study of a Physical Activity Promotion Intervention for At-Risk Patients in Primary Care

**DOI:** 10.2196/29035

**Published:** 2022-06-29

**Authors:** Keegan Knittle, Sarah J Charman, Sophie O'Connell, Leah Avery, Michael Catt, Falko F Sniehotta, Michael I Trenell

**Affiliations:** 1 Faculty of Social Sciences University of Helsinki Helsinki Finland; 2 Population Health Sciences Institute Newcastle University Newcastle upon Tyne United Kingdom; 3 Translational and Clinical Research Institute Faculty of Medical Sciences Newcastle University Newcastle upon Tyne United Kingdom; 4 Newcastle upon Tyne Hospitals National Health Service Foundation Trust Newcastle upon Tyne United Kingdom; 5 University Hospitals of Leicester National Health Service Trust Leicester United Kingdom; 6 School of Health & Life Sciences Teesside University Middlesbrough United Kingdom; 7 National Innovation Centre for Ageing Newcastle University Newcastle upon Tyne United Kingdom

**Keywords:** primary care, physical activity, cardiovascular disease, prevention, internet-based intervention, motivational interviewing, self-regulation

## Abstract

**Background:**

Physical activity (PA) can reduce cardiovascular disease (CVD) risk factors, and although primary care settings offer a large reach to promote PA and reduce CVD risk, primary health care professionals may lack self-efficacy and tools to effectively promote PA in practice. Movement as Medicine for CVD Prevention is a suite of 2 theory-based, web-based behavioral interventions—one for health care professionals and one for patients—which may offer a pathway for promoting PA and reducing CVD risk in primary care.

**Objective:**

This study aims to examine the feasibility and possible effects of Movement as Medicine for CVD Prevention.

**Methods:**

This nonrandomized pilot study recruited participants from primary care organizations in the Northeast of England. Enrolled health care professionals followed a theory-based, web-based course on PA counseling and motivational interviewing techniques. After the course, health care professionals delivered behavior change consultations based on motivational interviewing to inactive individuals with >20% risk of developing CVD within 10 years. Patients were then given access to a website based on self-determination and self-regulation theories, which targeted increased levels of PA. Outcomes were assessed at baseline and after 3 months, and patient data were analyzed on an intention-to-treat basis in a multiple imputation data set.

**Results:**

Recruitment rates of primary care organizations fell below expectations. A total of 11 health care professionals from 3 enrolled primary care organizations completed the web-based course and reported increases in important theoretical determinants of PA promotion in practice (eg, self-efficacy, Cohen *d*=1.24, 95% CI 0.67-1.80; and planning, Cohen *d*=0.85, 95% CI −0.01 to 1.69). A total of 83 patients were enrolled in the study, and 58 (70%) completed both the baseline and 3-month assessments. Compared with baseline, patients had higher levels of objective (Cohen *d*=0.77, 95% CI 0.13-1.41) but not subjective (Cohen *d*=0.40, 95% CI −0.03 to 0.83) moderate to vigorous PA at 3 months. Patients also reported higher levels of the PA determinants of intention, self-efficacy, intrinsic motivation, and action planning and action control at 3 months (effect sizes ranged from Cohen *d*=0.39 to 0.60).

**Conclusions:**

The Movement as Medicine for CVD Prevention intervention seems to have the potential to improve patient PA behaviors and important determinants of health care professionals’ PA promotion practices. However, the recruitment rates of primary care organizations in this study were low and would need to be increased to examine the efficacy of the program. This study offers several insights into improving the feasibility of this primary care PA promotion pathway.

**Trial Registration:**

ISRCTN Registry ISRCTN14582348; http://www.isrctn.com/ISRCTN14582348

## Introduction

### Background

Cardiovascular disease (CVD) accounts for approximately one-third of all deaths in the United Kingdom and places a substantial economic burden on the UK National Health Service (NHS), with costs estimated at £14 (US $18.3) billion and rising [1]. Epidemiological and experimental studies have established strong links between low levels of occupational and leisure time physical activity (PA) and an increased risk of CVD [2-5], and this is supported by findings from a recent meta-analysis [6], which found significant associations between moderate occupational and leisure time PA and CVD risk in both men and women. In addition, a wealth of evidence demonstrates that participation in PA is associated with improvements in metabolic risk factors for CVD and CVD-related mortality [5,7,8].

PA affects the main risk factors for the development of CVD, including decreases in low-density lipoprotein [9] and maintenance of normal glucose tolerance [10]. In addition, there are protective effects resulting from weight loss or maintenance [11] and its effect on blood pressure in physically active individuals. There are negative physiological responses associated with a lack of PA and high levels of sedentary behavior [12,13]. Research has shown that prolonged sedentary behavior produces adverse effects on the cardiovascular system, involving cardiac function, stroke volume, cardiac output, heart rate, thromboembolic events [14,15], and glucose intolerance [16]. Taken together, these findings indicate that population-level increases in PA and reductions in sedentary behavior could be vital in reducing CVD incidence and mortality.

The importance of increasing PA and reducing sedentary behavior to prevent noncommunicable diseases has been highlighted by the Chief Medical Officers of England, Scotland, Wales, and Northern Ireland in the *Start Active, Stay Active* document [17]. The UK Department of Health currently recommends that adults should accumulate a minimum of 150 minutes of moderate-intensity PA each week to achieve tangible health benefits [17]. However, despite the well-known CVD-related and general benefits of PA, only a small percentage of adults meet these government guidelines, with >60% of men and >70% of women in England being insufficiently active to benefit their health [18].

At present, low levels of PA at the population level are partially addressed through primary care–based screening programs, including the NHS Health Checks program in the United Kingdom. Such health check programs screen for risk factors, including low PA, that contribute to the incidence of CVD and type 2 diabetes and then signpost individuals to appropriate interventions. For example, when low PA is identified during a health check, patients are signposted to interventions to increase PA. However, the PA interventions offered to individuals in these primary care settings rarely match the existing evidence on optimal methods for increasing PA behavior, and PA promotion is not a primary aim of primary care–based screening programs. Although PA prescription and advice giving are the most used methods of promoting PA in primary care settings [19], there is considerable evidence that these methods do not create lasting increases in PA [20,21], with up to 26 people needing to receive PA advice to meet the recommended levels of PA 6 months later [22]. In addition, even these minimal advice-giving approaches to PA promotion are only delivered to a small percentage of individuals for whom PA interventions are warranted [23,24], meaning that existing PA promotion interventions in primary care are suboptimal and delivered too infrequently.

The problems with PA promotion in practice may reflect deficits in the knowledge, skills, or motivation levels of health care providers (HCPs) regarding the delivery of effective behavior change interventions. Existing evidence indicates that interventions to increase PA are most likely to succeed when they include behavior change techniques (BCTs) derived from the Self-Regulation Theory [25], including self-monitoring, goal setting, action planning, feedback, and problem-solving. However, many HCPs working in primary care cite a lack of adequate training on how to deliver such self-regulatory interventions as a barrier to implementation [26]. Furthermore, HCPs may face additional barriers that limit the extent to which they offer PA promotion interventions to patients. For example, they may not be motivated to deliver PA promotion interventions in the first place; they may think that PA promotion is unimportant or not part of their role; or they may perceive barriers or conflicting priorities that prevent them from promoting PA, even if they are motivated to do so [27]. Helping HCPs overcome these barriers is crucial to ensuring that HCPs can deliver effective PA interventions in primary care.

As research from high-income countries indicates that approximately 70% to 80% of adults visit their general practice at least once a year [28] and that many individuals are willing to discuss changes in health behaviors with their primary health care professionals [29], primary care offers great potential for population-level PA promotion. To make full use of this reach, primary care HCPs need to have the necessary knowledge and skills to effectively target PA, be sufficiently motivated to do so, have sufficient self-efficacy for promoting PA, and have evidence-based tools at their disposal to assist patients as they attempt to increase their PA behavior. To this end, our group developed the Movement as Medicine for CVD Prevention (MaMCVD) program: a 2-tiered suite of behavior change interventions that aim to increase PA and thereby mitigate CVD risk.

Briefly, MaMCVD is a new PA promotion care pathway that comprises 2 theory-based behavior change interventions. The first intervention was delivered to health care professionals (eg, general practitioners [GPs], practice nurses, health care assistants, and health improvement practitioners) and is based on the Health Action Process Approach (HAPA) [30] and Self-Determination Theory (SDT) [31]. It aims to foster HCPs’ self-efficacy and motivation for promoting PA in clinical practice and equip them with the knowledge and skills necessary to deliver behavior change interventions beyond traditional advice-giving and prescriptive approaches. The second intervention was delivered to patients and is based on the HAPA [30], SDT [31], and Self-Regulation Theory [32]. The patient intervention included techniques derived from motivational interviewing (MI) to increase patients’ motivation for behavior change during primary care consultations and provided patients with a set of web-based tools with which they could self-regulate their efforts to become more physically active.

### Aims

This study aims to examine the feasibility of MaMCVD as a PA promotion pathway in primary care settings, establish rates of recruitment and retention, and provide effect size estimates to potentially inform power calculations for a definitive randomized controlled trial (RCT). These aims are in line with phases 1 and 2 of the Medical Research Council framework for the development and evaluation of complex interventions to improve health [33].

## Methods

### Study Design

This nonrandomized pilot feasibility study aimed to examine a newly developed PA care pathway for individuals with an increased risk of developing CVD and involved data collection at both HCP and patient levels. The protocol was registered (ISRCTN14582348) and received approval from the Newcastle and North Tyneside 1 Research Ethics Committee (reference 14/ES/0032). All methods were performed in accordance with the relevant guidelines and regulations. Recruitment for the study began in June 2014, and the final study data were collected in May 2015.

The original protocol for this study specified an RCT; however, this changed to a nonrandomized trial because of difficulties in recruiting primary care organizations. In addition, the length of the study was shortened, and the primary outcomes were altered to better reflect the nature of this study as a feasibility trial. Full details of these changes are available in the trial registration record (ISRCTN14582348).

### Setting and Participants

#### Overview

The study took place within primary care organizations typically tasked with delivering NHS Health Checks in the North of England (eg, general practices and NHS Foundation Trusts) and recruited both HCPs and patients. The intervention for HCPs was delivered via the internet, and the patient intervention was delivered face-to-face by the MaMCVD-trained HCPs in primary care settings and subsequently via a web-based platform.

#### Inclusion and Exclusion Criteria

To be included in the study, primary care organizations were required to meet the following inclusion criteria: willing to follow the intervention, committed to participating for up to 12 months depending on patient recruitment rates, at least 2 HCPs from the organization were willing and able to take part, capable and willing to identify and recruit patients meeting the eligibility criteria, and able to provide researchers with patient contact details to facilitate the mailing of questionnaires and accelerometers for data collection after a patient has provided informed consent. Individual HCPs were eligible for participation if they were normally part of a follow-up visit after an NHS Health Check (eg, nurses, physicians, allied health professionals, health visitors, and community health workers) and were willing to comply with the study protocol and complete the web-based training course for continuing professional development (CPD) credit.

Patients who had been identified as physically inactive with the General Practice PA Questionnaire [34] and who had at least a 20% risk of developing CVD in the next 10 years based on the QRISK2 cardiovascular risk algorithm [35] were eligible for inclusion in the study. In addition, patients needed to be aged between 18 and 75 years, have the capacity to provide informed written consent, be able to speak and read English without the support of an interpreter, have access to the internet, and be cleared to partake in PA according to the Physical Activity Readiness Questionnaire (PAR-Q) [36].

### Study Interventions

The development of the MaMCVD primary care PA promotion pathway was informed by our group’s previous work in developing a primary care PA promotion pathway for people with type 2 diabetes [37]. The core of MaMCVD was an integrated website with separate areas for HCPs and their patients, described in detail in the following sections.

#### Interventions for Health Care Professionals

After providing informed consent and completing baseline measures, health care professionals received a 20-minute motivational interview to elicit their reasons and motivations for potentially promoting PA with patients who have an increased risk of CVD and help strengthen their intentions to deliver behavior change interventions to such patients. These MI sessions took place in the HCPs’ workplaces and were delivered by a trained MI trainer (KK). The BCTs [38] used in each phase of the MaMCVD intervention for HCPs are outlined in Table 1.

**Table 1:** Description of the BCTs^a^ delivered over the course of the study to health care professionals.

BCT	Before following the MaMCVD^b^ course	As part of the web-based MaMCVD course	After following the MaMCVD course	After delivering the MaMCVD consultations^c^
Motivational interviewing	✓			
Provide normative information about others’ consultation behaviors		✓		
Information on where or when to perform behaviors		✓		
Instruction on how to perform behaviors		✓		
Model or demonstrate the behaviors		✓		
Teach to use prompts or cues		✓		
Prompt practice		✓		
Provide reward contingent on completing the course			✓	
Provide feedback on performance				✓
Barrier identification or problem-solving				✓

^a^BCT: behavior change technique.

^b^MaMCVD: Movement as Medicine for Cardiovascular Disease Prevention.

^c^BCTs in this column were delivered after the effects of the intervention for health care providers were assessed. In other words, audit and feedback procedures were implemented to improve the quality of the motivational interview sessions delivered to patients during the study.

After the MI session, HCPs were given access to a web-based course comprising 11 interrelated modules on topics such as PA and sedentary behavior in relation to CVD, the processes involved in behavior change, and study-related procedures. Table 2 provides additional information about the topics of the modules, each of which was reviewed by experts in changing the clinical behaviors of HCPs. The course aimed to equip HCPs working in primary care with the knowledge and skills necessary to effectively promote PA among their patients and included static information about several key elements of MI [39] and self-regulation interventions [32]. Textual information, interactive info boxes, video demonstrations, and quiz questions aimed to prepare HCPs to confidently deliver the behavior change intervention to participating patients and help them overcome barriers to promoting PA in their day-to-day practice (ie, increase their self-efficacy for promoting PA). In total, the 11 modules took each practitioner approximately 4 hours to complete. HCPs were given 4 weeks to work their way through the intervention, and their log-in provided unlimited and ongoing access to the intervention content, including the web-based tools available to patients. After successful completion of the web-based course, including passing (≥80% correct) a 20-question multiple-choice end-of-course assessment, the HCPs received a certificate and CPD credit. HCPs were subsequently informed that they could begin delivering the MaMCVD intervention to their patients.

Initial sessions taking place between HCPs and patients who had both consented to have the consultations recorded were audio recorded. Recordings were then returned to the research team, who coded and assessed each consultation for clinician skill in delivering the intervention per protocol. From these recordings, researchers generated delivery feedback reports to assist the HCP in further improving their skills in MI and delivering behavior change interventions. This audit and feedback process highlighted areas of the consultation that went particularly well and were in line with what they had learned in the web-based course, as well as areas of the consultation that were delivered in a way that was not adherent to what was taught in the web-based course. For the areas of the consultation that were not delivered in line with the protocol, HCPs were prompted to identify what went wrong and think about ways of preventing similar mistakes from happening in future MaMCVD consultations. The feedback was provided in written form and was, in some cases, followed up by a telephone call or in-person visit to highlight or further explain points from the written feedback. To aid recall of the consultations upon which feedback reports were created, the research team aimed to provide HCPs with delivery feedback reports within 1 week. Therefore, HCPs were asked to stagger initial sessions with patients at 2-week intervals so that feedback could be delivered before the next set of sessions took place.

**Table 2:** Descriptions of the contents and duration of the modules included in the Movement as Medicine web-based course for health care professionals.

Module number	Title	Description	Duration (min)
1	Introduction to Movement as Medicine for CVD^a^ Prevention	Video overview of course contents	5
2	Background of CVD	Information about CVD prevalence, mortality, costs associated with the treatment of CVD, and costs of CVD to the UK economy or NHS^b^	15
3	PA^c^ and CVD	Information detailing the relationship between PA frequency or intensity and common CVD risk factors	15
4	Sedentary behavior and CVD	Information detailing the relationship between sedentary behavior and CVD risk factors	10
5	An introduction to the process of behavior change	Outline 2 distinct stages of behavioral change: motivation and action	10
6	Fostering motivation for change	Introduction of the importance of change talk	20
7	Clinical skills—asking	Learn skills to elicit change talk from patients	20
8	Clinical skills—listening	Learn ways to reflect change talk back to patients	20
9	Clinical skills—informing	Learn alternatives to information provision, such as using the elicit-provide-elicit structure	20
10	Use of patient self-regulation tools	Provides rationale and evidence for the effectiveness of self-regulation approaches to behavior change Provides full access to the web-based self-regulation materials available to patients in the trial, including walk-throughs and demos	30
11	Practical information for the Movement as Medicine trial	Information about recruitment, timing of patient contacts, and feedback to be received about delivery	15

^a^CVD: cardiovascular disease.

^b^NHS: National Health Service.

^c^PA: physical activity.

#### Interventions for Patients

Over the course of the intervention, patients were scheduled to have one face-to-face and one telephone contact (of up to 30 minutes each) with their Movement as Medicine–trained HCP at baseline and 2 months, respectively. In addition, they were provided access to web-based behavior change resources and tools developed in line with the HAPA [30], SDT [40], and Self-Regulation Theory [32]. The BCTs [38] included in this intervention are enumerated on a per-component basis in Table 3, and screenshots of many web-based components are available in Multimedia Appendix 1. The patient intervention would include a maximum of 1 hour of contact time with an HCP, and participants could spend as much or as little time as they desired using the web-based behavior change resources and tools.

During a patient’s first MaMCVD consultation, HCPs applied the key elements of MI in health care settings that they learned during the web-based MaMCVD course [39]. The goal of this initial consultation was to help patients form an intention to increase their PA behavior, and therefore, it also included techniques designed to target theoretical predictors of intention by fostering self-efficacy [41,42], addressing outcome expectancies and autonomous motivation for change, and providing information and advice only where necessary and in a way that supports patient autonomy and control [43]. During the session, HCPs obtained information about how PA fits into the patient’s life, explored times in the past when the patient was more physically active, identified and weighed the pros and cons of potentially taking more PA, and helped patients work through this ambivalence. HCPs also aimed to create and appropriately respond to patient utterances of change talk and worked to elicit patients’ motivations for change and long-term (outcome) goals. During the consultations, HCPs made efforts to adhere to the spirit of MI and use MI-adherent behaviors such as obtaining permission before providing advice or information, affirming the patient, emphasizing the patient’s control, and supporting the patient with compassion or sympathy [39]. In addition, HCPs were instructed to avoid MI nonadherent behaviors such as advising without permission; confronting the patient; or directing the patient by giving orders, commands, or prescriptions [39], as a positive balance between MI-adherent and MI-nonadherent behaviors has been shown to predict favorable outcomes in consultations about PA behavior changes [44,45].

**Table 3:** Description of the BCTs^a^ delivered over the course of the MaMCVD^b^ intervention for patients.

BCT	Consultation 1 (in person)	In the web-based MaMCVD materials	Consultation 2 (telephone)
Motivational interviewing	✓		✓
Prompt focus on past success	✓		✓
Individualized information on consequences of PA^c^	✓	✓	✓
Outcome goal setting	✓	✓	
Information on general consequences of PA		✓	
Behavioral goal setting		✓	
Action planning		✓	
Prompt self-monitoring of behavior		✓	
Barrier identification and problem-solving		✓	✓
Prompt review of behavioral goals		✓	✓
Prompt review of outcome goals		✓	✓
Relapse prevention or coping planning		✓	✓
Provide feedback on performance		✓	✓
Provide info on when and where to perform PA		✓	
Provide rewards contingent on behavior		✓	
Provide rewards contingent on progress		✓	
Teach to use prompts and cues		✓	
Use of follow-up prompts			✓

^a^BCT: behavior change technique.

^b^MaMCVD: Movement as Medicine for Cardiovascular Disease Prevention.

^c^PA: physical activity.

At the end of this initial session, a time and date for the follow-up telephone call were planned, and the HCP provided each patient with a pedometer, an activity log, and a unique log-in and password for the MaMCVD web-based behavior change tools. The primary aim of this patient website was to help people translate their intentions to be more physically active into actual PA behavior [46]. However, to accommodate patients who did not form an intention to increase their PA after their initial face-to-face consultation, the web-based behavior change tools also included techniques to promote intention formation.

Upon logging into the MaMCVD patient website, patients were directed to the *Decision Dashboard* preintentional portion of the site, which contained information pages and interactive assessments of patients’ pros and cons of change and current PA levels. The information pages described the benefits of PA to health and well-being, government recommendations for PA, places in the community where participation in PA or sports was possible, and information about how to enjoy PA safely. The assessment of pros and cons took in users’ own ideas about PA and provided tailored feedback about their intention strength. The assessment of current PA accepted user-recorded levels of PA based on 1 week of self-monitoring via the activity log and pedometer provided in the first session and provided tailored, autonomy-supportive feedback based on a comparison of patients’ logged PA and current government guidelines for PA. Patients were free to explore these segments in any order they wished and could, at any time, click on a button within the *Decision Dashboard* to indicate to the system that they wished to launch the *Activity Dashboard* (ie, he or she had formed an intention to become more physically active).

After launching the *Activity Dashboard*, patients were asked to specify their motivation for wanting to become more physically active (ie, set an outcome goal). Patients who had difficulty specifying their motivation were offered a motivation assessment tool based on the Exercise Motivations Inventory [47]. In the motivation assessment tool, patients stated the strength of their desires to achieve the several outcomes that PA can produce (eg, vitality, health, and social) and subsequently received tailored feedback about the outcomes on which they scored the highest.

After choosing a motivation, users proceeded to the postintentional *Activity Dashboard* portion of the website, which contained several BCTs derived from Self-Regulation Theory (ie, self-monitoring, feedback, goal setting, action planning, and problem-solving). This set of techniques has been shown to play a vital role in increasing PA levels in previous research [25,48]. To establish a baseline level of PA, if they had not done so already in the *Decision Dashboard* portion of the website, patients were asked to self-monitor their behavior for a 1-week period. These data could be inputted by entering the duration and intensity of PAs they had engaged in or by entering the daily step counts from their pedometer. The system converted any activities entered into steps using metabolic equivalent values [49], which allowed for the entry of activities that could not be recorded by a pedometer (eg, swimming and cycling) and for the presentation of all activities using a common metric.

After 1 week of tracking to establish a baseline level of PA, patients were prompted to set a week-long PA goal for total steps or average steps per day. To reduce the risk of users failing to achieve their goals, as this could undermine self-efficacy and motivation [50], patients received visual feedback on the assumed difficulty of their new goal based on a comparison with their average activity level over the past 4 weeks. This gradual method of PA growth aimed to increase self-efficacy in PA [41].

After setting a goal, individuals were prompted to plan specific activities, times, durations, locations, and intensities of PA, which would lead them to achieve their weekly goal. Patients could also indicate whether they would like to receive reminders about their planned activities via email.

The *Activity Dashboard* also contained a problem-solving tool (based on the volitional help sheets of Armitage and Arden [51]) with which users could identify personally relevant barriers to PA participation, view common ways to overcome each barrier, brainstorm their own solutions, and make explicit links between their chosen barriers and solutions.

In engaging with the Movement as Medicine website, patients could use as many or as few of the self-regulation resources as they deemed necessary. Patients received rewards in the form of web-based badges for engaging with various aspects of the website, increasing PA in consecutive weeks, logging into the site in consecutive weeks, achieving their PA goals, achieving weekly or lifetime milestones, and meeting government guidelines for PA. In addition, users were prompted by email to revisit the site after a prolonged time between log-ins and were given tailored advice if they failed to achieve goals in successive goal periods.

After 2 months from the initial MI consultation (+1 week or –1 week), the patients received a follow-up phone call from their HCP to discuss their progress to that point. The structure of this consultation was flexible and tailored to reflect the extent to which each patient had formed an intention or engaged with the web-based self-regulation materials. For patients who still had not formed an intention to increase PA, the consultation would, in many ways, reflect the initial MI consultation, focusing on motivation for change and long-term outcome goals. For patients who had formed an intention and had engaged with the self-regulation materials, the call focused on providing technical assistance with the website, helping the patient talk through and overcome barriers to PA, and providing support for the patient’s efforts. Regardless of the content, phone consultations were to be conducted in an MI-adherent way to continue to foster motivation for sustained behavioral change.

### Outcome and Process Measures

#### Health Care Professional Measures

##### Overview

To assess the effects of the MaMCVD web-based course on HCPs’ likelihood of effectively delivering PA promotion sessions in practice, we assessed important theoretical predictors of this outcome at baseline and after the completion of the course. These constructs included knowledge of the relationships between CVD and PA; self-efficacy, outcome expectancies, and intention to promote PA to patients in practice [52]; and autonomous motivation for delivering PA behavior change interventions to patients [53].

##### Evaluation of the Web-Based MaMCVD Course Materials

To obtain information about the acceptability of the web-based course, interviews were conducted with each HCP upon completion of the course to identify aspects of the course that required modification and were most or least useful, as well as to identify any technical problems encountered by HCPs while following the course. The interview topic guide is available in the Multimedia Appendix 1.

#### Patient Measures

Patient measures were assessed at baseline and 3 months. At both measurement points, the research staff mailed the patients an accelerometer and a guide for its use, a questionnaire booklet, and a stamped and addressed envelope to return the accelerometer and questionnaire pack to the research team.

##### PA Measures

Wrist-worn accelerometers (AX3, Axivity) [54] were used to capture 7-day monitoring of sedentary behavior and PA levels under free-living conditions. The AX3 is a triaxial accelerometer configured for sampling at 100 Hz [54]. The accelerometer was preprogrammed to start on disconnect and posted to the participant to start wearing the day after receiving the monitor, and instructions were provided on how to wear the accelerometer.

Accelerometer data were processed in R (R Foundation for Statistical Computing) [55] using the package GGIR [56]. The signals were inspected and corrected for calibration error [57]. The first and last hours of the measurement were excluded as they were expected to be influenced by the monitor distribution and collection procedure. Next, the average magnitude of wrist acceleration per 5-second epoch was calculated using the metric Euclidean Norm Minus One, as previously described [56]. The output from the metric Euclidean Norm Minus One is in milligram (1 mg=0.001 g=0.001×9.8 m/s^2^=0.001×gravitational acceleration) [56]. Monitor nonwear was detected as described previously [56] and replaced by the average accelerometer data at similar time points on different days of the measurement [58,59]. The imputation procedure used was effectively the same as taking the average of all available data weighted by the number of available data points per time of the day. In contrast, taking the plain average of all available data points would cause unequal weighting of periods within the 24-hour cycle and result in an unstandardized estimate of PA. The resulting time series was used to derive the time spent in the acceleration categories per day. Moderate to vigorous PA (MVPA) was calculated using a ≥100 mg cutoff, and the outcome was displayed as an average for the week [60]. The average most and least-active 5-hour periods of each day were also calculated in milligrams. Only patients with at least 5 days of valid measurement (ie, at least 16 hours of wear time in a 24-hour period) in each assessment period were included in the analyses [61].

Patients’ subjective PA levels were assessed using the short version of the International PA Questionnaire [62]. The primary outcome of interest from this questionnaire was the total MVPA (minutes per week). Self-reported leisure time MVPA (minutes per week) and sitting time (hours per day) are also reported.

##### Theoretical Predictors of PA

Predictors of PA derived from the HAPA [30] and SDT [40] were assessed among patients via postal questionnaires delivered at baseline and 3 months. The HAPA questionnaire [63] assessed risk perceptions (both absolute and relative), outcome expectancies, action planning, action control, and intention for PA with items using 4-, 5- and 7-point Likert scales. Means of the items were used to create the total score for each scale.

Self-efficacy for PA was assessed using the 18-item Exercise Self-efficacy Questionnaire developed by Bandura [64]. Each item presents a situation in which it may be difficult to engage in PA (eg, when busy and in bad weather) and allows participants to rate the likelihood that they could be physically active from 0 (not at all likely) to 10 (certainly). The mean of 18 items was used as the total self-efficacy score.

The Behavioral Regulation in Exercise Questionnaire was used to measure the continuum of behavioral regulation for PA [53]. It is a 19-item questionnaire that assesses intrinsic, identified, introjected, and external regulatory styles for exercise, with responses given on a 5-point Likert scale. The scales were calculated by taking the mean of the items within each regulatory style.

Depressive symptoms were assessed using the Patient Health Questionnaire [65], a validated brief self-report inventory commonly administered in primary care, which addresses the presence and severity of the 9 diagnostic criteria for major depressive disorder from the Diagnostic and Statistical Manual of Mental Disorders [66].

Control beliefs about developing CVD were assessed using a modified 6-item version of the Brief Illness Perceptions Questionnaire [67]. Patients’ feelings of control from both personal and treatment-related sources were assessed on an 11-point Likert scale (0-10).

##### Use of Movement as Medicine Intervention Materials

Each patient’s progress and interactions with the web-based MaMCVD intervention were anonymously logged by the system to identify the point at which patients proceeded to the postintentional website components and monitor the extent to which each patient viewed the information pages and engaged with the behavior change tools found on the website.

### Trial Procedures

#### Practice Recruitment

Primary care organizations from the Northeast of England were approached to participate through meetings of clinical research networks within the NHS, as well as through local and regional meetings of general practice managers. In these meetings, researchers presented a rationale for the study, an outline of its procedures, and reimbursement schemes available to help practices cover the costs of treatment and recruitment. Primary care organizations were actively followed up by phone or email after these meetings, and those that responded favorably and expressed a capability to enroll at least two health care professionals in the study were included in the study. Recruitment of primary care organizations was planned to continue until 9 primary care organizations had been recruited or after 4 months of active recruitment.

HCPs from the recruited primary care organizations were given information sheets and asked to provide informed consent for the study. Participation was voluntary, and the HCPs were free to withdraw from the study at any time. In cases where HCPs withdrew from the study after contributing data, data already collected were included in the study report unless they were specifically requested to be removed.

#### Patient Recruitment

Patients were recruited through 2 possible streams of enrollment, and enrollment began after 2 HCPs from a participating primary care organization consented to participate in the study. In the first stream, a member of staff from each participating primary care organization accessed electronic patient record databases and selected a random sample of 200 or 400 patients (depending on the practice list size) who fulfilled the inclusion criteria and had at least a 20% risk of developing CVD over the next 10 years, as assessed by the QRISK2 algorithm [35]. The research staff then mailed a recruitment pack to each of these randomly selected eligible patients, which included an invitation letter, a patient information sheet, the PA Readiness Questionnaire [68], an informed consent form (all printed on paper headed with the details of that primary care organization), and a prepaid return envelope. In the second stream, patients who had recently undergone an NHS Health Check and who met the inclusion criteria were given the same abovementioned recruitment pack, with Movement as Medicine described as a brief PA intervention that fulfills Public Health England’s Best Practice Guidance for the NHS Health Checks program [69]. Patient recruitment was planned to continue for 4 months at each site or until the overall target of 198 patients was reached. The target sample size of 198 was based on sample size calculations for multiple regression analyses to investigate processes within the intervention (α=.05; power=0.80; anticipating a medium effect size with 10 independent variables). Initial calculations in G*Power [70] indicated that 118 patients were needed to detect effects; however, after accounting for a potential dropout of approximately 20% and cluster effects based on primary care organization and HCP, the necessary sample size rose to 198 patients, recruiting 22 patients from each of the 9 recruited primary care organizations. This sample size was not achieved within the 4-month window of recruitment.

Patients interested in taking part were asked to return the written informed consent form and the completed PAR-Q to the primary care organization in the prepaid envelope. If the patients failed the PAR-Q form by answering *yes* to any of the questions, they were required to obtain written approval from their GP before enrolling in the study. Upon arriving for their face-to-face consultation at the beginning of the study, patients had the chance to ask further questions and signed the consent form again in the presence of a member of staff to ensure that patients fully understood the study procedures. Patients who did not respond to the initial recruitment pack mail within 1 month were sent a second recruitment pack. If no response was received after this, no further efforts were made to recruit that patient. Primary care organizations continued to contact eligible patients in this manner for 4 months.

Patients were made aware that their participation in the study was entirely voluntary and that they could withdraw at any time without providing a reason and without their legal rights or health care being affected. In cases where participants withdrew from the study after contributing data, these data were used in the analyses unless they specifically requested that they be removed.

Patients who dropped out of the study were mailed a postcard with 3 very short questions to obtain their reasons for dropping out. The return of this postcard was entirely at the patients’ discretion and was intended to gather important information that could be used to alter the program and procedures to reduce the likelihood of future dropouts for the same reasons.

### Statistical Analyses

Paired *t* tests (2-tailed) in SPSS (SPSS Inc) were used to compare the baseline and follow-up levels of HCP outcomes. Multiple imputation (*i=*5) was undertaken to account for missing patient data at the 3-month follow-up. The imputation model used baseline demographic information and baseline and follow-up data of all study outcomes to predict the missing follow-up values of all study outcomes. Paired *t* tests were used to compare baseline and follow-up levels of patient outcomes in the pooled intention-to-treat data set. Cohen *d* effect sizes, and 95% CIs were also calculated to estimate the potential efficacy of the interventions and inform the sample size and power calculations for subsequent testing of this intervention [71]. Qualitative data from interviews with HCPs were examined using content analysis.

### Ethics Approval and Consent to Participate

A favorable ethical opinion was granted by Newcastle and North Tyneside 1 NHS Research Ethics Committee (reference 14/ES/0032). Informed consent was obtained from all research participants.

### Availability of Data and Materials

The data sets generated and analyzed during this study are not publicly available, as ethical approval for the sharing of data was not sought or obtained; however, these are available from the corresponding author on reasonable request.

## Results

### Recruitment

A total of 128 primary care organizations in the Northeast of England were approached for participation in the study. Of the 128 approached organizations, 5 (3.9%) primary care organizations (ie, n=4, 80% general practices and n=1, 20% community health organization) were willing to participate in the study. However, 50% (2/4) of the general practices dropped out of the study because of staff turnover and lack of available resources to administer the study.

**Figure 1:**
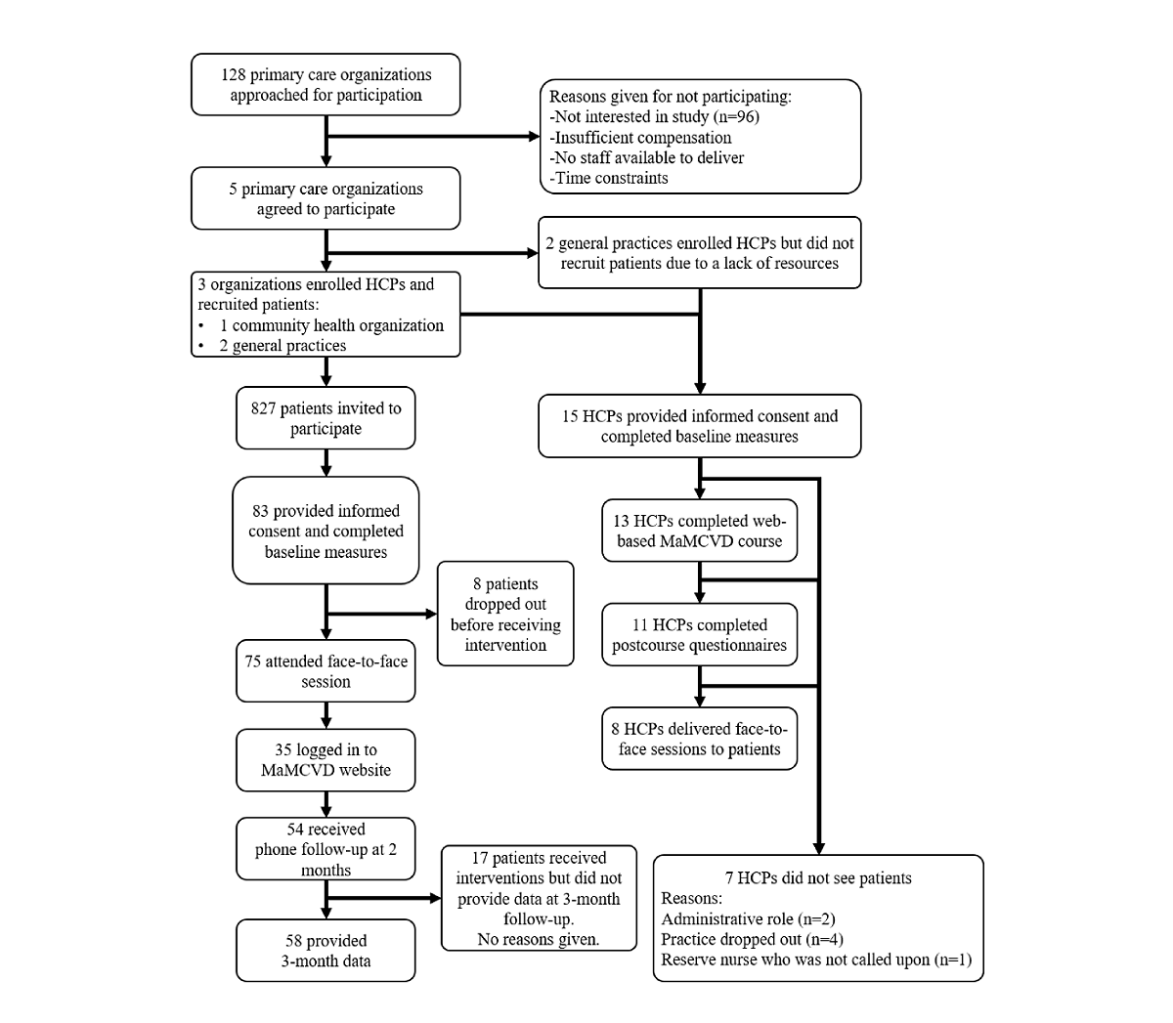
Flow of individuals through the study. HCP: health care provider; MaMCVD: Movement as Medicine for Cardiovascular Disease Prevention.

Within the 3 remaining primary care organizations, recruitment packs were mailed to 827 patients. Of these 827 patients, 84 (10.2%) provided informed consent to participate in the study, with 83 (10%) subsequently completing baseline measures. Recruitment rates were 7%, 11%, and 12% across the 3 included primary care organizations. Detailed information on the flow of primary care organizations, HCPs, and individuals throughout the study can be found in Figure 1.

### HCP Characteristics

A total of 11 HCPs completed the web-based course and postcourse questionnaires. Of these 11 HCPs, 7 (64%) were female, with a mean age of 38.8 (SD 9.9; range 28-52) years. Enrolled HCPs were PA specialists (5/11, 46%), practice nurses (3/11, 27%), health care assistants (2/11, 18%), and GPs (1/11, 9%) and had an average of 7.1 years in their current role. Of the 11 HCPs, 7 (64%) had previously received some form of training in behavior change methods or BCTs.

### Acceptability of Web-Based MaMCVD Course Materials and Changes in HCP Outcomes

After completing the web-based course, the HCPs reported increases in self-efficacy for promoting PA in practice and for having concrete plans of how and where to promote PA in practice. Smaller increases in habit were also reported, whereas reported changes in attitudes toward PA promotion, intention for PA promotion, and goal conflict were negligible. Quantitative results for the HCP outcomes are presented in Table 4.

**Table 4:** Effects of Movement as Medicine for CVD^a^ Prevention on health care provider outcomes (N=11).

Outcome	Baseline, mean (SD)	Postcourse period, mean (SD)	*P* value^b^	Cohen *d* (95% CI)
Self-efficacy	3.91 (0.82)	5.03 (0.98)	.002	1.24 (0.67 to 1.80)
Attitudes	6.82 (0.34)	6.86 (0.26)	.69	0.13 (−0.40 to 0.66)
Intention	5.91 (0.77)	5.86 (1.23)	.91	−0.05 (−0.65 to 0.56)
Planning	4.48 (1.42)	5.45 (0.75)	.05	0.85 (−0.01 to 1.69)
Habit	4.93 (1.62)	5.55 (1.33)	.04	0.42 (0.02 to 0.80)
Goal conflict^c^	3.68 (1.33)	3.59 (1.53)	.76	−0.06 (−0.46 to 0.34)

^a^CDV: cardiovascular disease.

^b^*P* values reported are for paired *t* tests and are not corrected for multiple comparisons. Instead, we refer readers to the reported effect size estimates and CIs.

^c^For this outcome, a negative effect size indicates a favorable result of the intervention.

Qualitative interviews with HCPs indicated broad acceptability of the web-based course, with an appreciation for the demonstration videos of MI techniques and several of the interactive educational elements. Some HCPs were unable to access the web-based course from the computer workstations in their primary care practice because of outdated browsers still being in use. In these cases, HCPs completed the web-based course using their home computer or tablet.

### Patient Characteristics

Of the 83 patients who provided data at baseline, 44 (53%) were female, and the average age was 57.5 (SD 10.2) years; 62 (75%) were married or cohabitating, and 41 (49%) were in part- or full-time employment, whereas 40 (48%) were unemployed or retired. Of the 83 patients, 15 (18%) had not completed high school or equivalent vocational education, whereas 25 (30%) had completed a university degree. Approximately 70% (58/83) of participants completed the 3-month follow-up measures, and there were no significant differences between patients who dropped out of the study and those who completed both baseline and follow-up assessments.

### Changes in Patient Outcomes

Analyses of intention-to-treat data revealed a significant increase of 9.6 minutes per day in objectively measured MVPA from baseline to follow-up (effect size Cohen *d=*0.77). Participants also reported favorable increases in self-reported total MVPA and MVPA during leisure time, as well as small reductions in sedentary behavior, all with effect sizes >Cohen *d=*0.44. Of the psychological variables assessed, patients reported favorable changes in self-efficacy for PA, PA action planning, action control, and intrinsic motivation for PA, with effect sizes between Cohen *d=*0.46 and Cohen *d=*0.60. The effect sizes for most of the other outcomes were <Cohen *d=*0.30. Table 5 provides for the means, SDs, and effect sizes with CIs.

**Table 5:** Effects of the Movement as Medicine for CVD^a^ Prevention intervention on patient outcomes.

Outcome		Baseline, mean (SD)	3 months, mean (SD)	*P* value^b^	Cohen *d* (95% CI)
**Objective PA^c,d^**					
	MVPA^e^ (minutes per day)	83.1 (36.5)	92.7 (38.3)	.02	0.77 (0.13 to 1.41)
	L5^f^	3.6 (0.7)	4.0 (2.5)	.32	0.32 (–0.31 to 0.94)
	M5^g^	39.7 (9.2)	43.9 (11.5)	.01	0.94 (0.29 to 1.60)
	ENMO^h^ (mg)^i^	20.9 (4.8)	22.4 (5.2)	.01	0.83 (0.19 to 1.48)
**Subjective PA**					
	IPAQ^j^ total MVPA (minutes per week)	318 (203)	349 (172)	.18	0.30 (–0.14 to 0.74)
	IPAQ leisure time MVPA (minutes per week)	36.5 (87.4)	58.2 (76.7)	.03	0.49 (0.05 to 0.93)
	Sitting time (hours per day)^k^	5.85 (2.81)	5.24 (2.43)	.05	–0.44 (–0.88 to 0.002)
**Determinants of PA from HAPA^l^**					
	Intention for PA	4.51 (1.24)	4.77 (1.09)	.09	0.39 (–0.06 to 0.84)
	Self-efficacy for PA	4.61 (1.78)	5.19 (1.76)	.01	0.59 (0.14 to 1.03)
	Action planning for PA	3.32 (1.84)	3.92 (1.67)	.01	0.60 (0.15 to 1.06)
	PA outcome expectancies	3.50 (0.71)	3.44 (0.64)	.51	–0.15 (–0.60 to 0.30)
	Perceived barriers to PA^k^	1.99 (1.04)	1.88 (1.10)	.52	–0.15 (–0.59 to 0.30)
	Action control for PA	1.50 (0.78)	1.81 (0.80)	.02	0.54 (0.09 to 1.00)
**Regulatory style**					
	Intrinsic motivation	2.08 (1.22)	2.36 (1.23)	.047	0.46 (0.01 to 0.91)
	Identified motivation	2.36 (0.99)	2.59 (0.97)	.05	0.45 (–0.001 to 0.90)
	Introjected motivation^k^	1.07 (0.95)	1.20 (0.89)	.35	0.21 (–0.23 to 0.65)
	External motivation^k^	0.49 (0.69)	0.61 (0.71)	.27	0.25 (–0.19 to 0.70)
	Amotivation^k^	0.47 (0.76)	0.48 (0.64)	.96	0.01 (–0.43 to 0.46)
**Illness perceptions**					
	Personal control	6.10 (1.71)	6.63 (1.54)	.39	0.20 (–0.25 to 0.64)
	Treatment control (PA)	7.88 (1.49)	7.24 (2.56)	.23	–0.27 (–0.72 to 0.17)
	Concern	6.42 (2.50)	5.90 (2.65)	.19	–0.30 (–0.75 to 0.15)
	Prevention comprehension	6.67 (2.15)	6.94 (2.12)	.54	0.14 (–0.31 to 0.58)
**Other outcomes**					
	Depressive symptoms^k^	5.95 (5.83)	4.99 (4.58)	.16	–0.32 (–0.77 to 0.13)
	Perceived CVD risk (relative)	3.08 (1.25)	3.45 (1.86)	.21	0.29 (–0.16 to 0.74)
	Perceived CVD risk (%; absolute)	48.7 (21.0)	44.2 (26.8)	.38	–0.21 (–0.69 to 0.26)

^a^CVD: cardiovascular disease.

^b^*P* values reported are for paired *t* tests using pooled multiple imputation data and are not corrected for multiple comparisons. Readers are instead referred to the reported effect sizes and 95% CIs.

^c^PA: physical activity.

^d^Objective physical activity data are for individuals with at least 5 days of valid accelerometer wear time at both baseline and 3-month assessment periods (n=40). All other outcomes are reported for individuals who completed both baseline and 3-month questionnaires (n=58).

^e^MVPA: moderate to vigorous physical activity.

^f^Average least active 5-hour period of each day in mg.

^g^Average most active 5-hour period of each day in mg.

^h^ENMO: Euclidean Norm Minus One.

^i^Average wrist acceleration.

^j^IPAQ: International Physical Activity Questionnaire.

^k^Lower scores are desirable for this outcome; thus, a negative effect size indicates a favorable result of the intervention.

^l^HAPA: Health Action Process Approach.

### Use of Web-Based Tools

Of the 75 patients who attended a face-to-face session and received log-in credentials for the patient website, 35 (47%) logged in at least once. Among these 35 individuals, 7 (20%) logged into the system weekly for at least 10 consecutive weeks, and the mean number of log-ins during the 3-month study period was 19.6 (mean log-ins per week 1.5). The most active user during the study period logged in 156 times or an average of 12 times per week. Further data on the use of individual components of the web-based intervention are presented in Table 6.

**Table 6:** Numbers of patients who used the web-based components of the Movement as Medicine intervention (n=35).

Component			Users,^a^ n (%)
**Motivation-focused components**			
	Weighing pros and cons tool	5 (14)	
	Motivation assessment tool	3 (9)	
	Indicated a decision to become more physically active	15 (43)	
**Self-regulatory components**			
	Set at least one physical activity goal	11 (31)	
	Logged some self-monitored physical activity	10 (29)	
	Made at least one physical activity action plan	4 (11)	
	Formulated at least one coping plan using the problem-solving tool	4 (11)	
	Used self-monitoring plus at least one other self-regulatory component	10 (29)	
	Used all self-regulatory components	3 (9)	

^a^Percentages indicate the proportion of individuals who logged into the patient website at least once (n=35) that used each component.

## Discussion

### Principal Findings

This nonrandomized pilot study assessed the feasibility of MaMCVD, a suite of 2 behavior change interventions for HCPs and patients to promote PA in primary care settings. MaMCVD was designed to provide HCPs with the skills required to increase motivation for PA among patients with CVD risk and help them address common barriers to promoting PA in primary care settings. In addition, it aimed to offer patients a set of theory- and evidence-based tools that they could use to self-regulate their efforts toward increasing PA and reducing CVD risk.

### Feasibility of the MaMCVD Program

Among the patients approached for participation in this study, recruitment and retention rates were in line with expectations; however, recruitment of primary care organizations fell below expectations. This is attributable to several factors, including a major reorganization of primary care service delivery within the NHS just before the commencement of this study. In 2013, primary care trusts were disbanded and replaced by clinical commissioning groups, which created uncertainty as to whether and how participation in research studies would be reimbursed [72]. In addition, as this study was funded by a local (as opposed to a national or international) organization, it was not eligible for adoption by the National Institute for Health Research (NIHR) clinical research network [73]. Adoption to the NIHR portfolio would have provided additional financial compensation and logistical assistance to primary care organizations for taking part [74] beyond the CPD training and logistical assistance provided in this study. During recruitment, conversations with research leads from several primary care organizations indicated insufficient compensation and a lack of available staff as the most common reasons for nonparticipation in the study. Identifying additional compensation possibilities for practices and ensuring study adoption by primary care research networks will be key to improving the recruitment of primary care organizations when testing MaMCVD in a larger RCT.

The 30% rate of patient dropout in this study is similar to that reported in studies testing other (web-based) PA interventions among individuals with chronic diseases [75]. Although we attempted to gather information about the reasons why participants dropped out of the trial by mailing a postcard to those who did, none of these postcards were returned to the study team, and we were unable to gather such information. In any full-scale trial of MaMCVD, it might be worth including efforts to improve study retention, especially if outcomes are examined with a longer follow-up period. This could potentially include offering patients a choice of intervention components based on their preference (eg, web only, face-to-face only, or both options). For HCPs, the potential perceived burden of both receiving and delivering an intervention within the same study, as well as the share of total working time study-related obligations take up, should also be considered.

The web-based tools that constituted part of the MaMCVD intervention for patients were not used by all the participants. Approximately half of the participants who attended an MI session logged into the patient website, which is somewhat lower than the uptake reported in other internet-based PA interventions [76]. This could potentially be attributed to whether and how a patient’s HCP introduced the website. As the study did not include any checks of whether patients received website log-in credentials from their HCP, it is possible that HCPs may have forgotten to deliver these to some patients. This element of intervention fidelity should be assessed in any further rollout of MaMCVD. In addition, audio recordings of MI sessions early in the trial revealed that some HCPs misinformed patients about the purpose of the patient website, referring to it simply as a place where patients could obtain additional information about PA. As much of the content of face-to-face MI sessions was already informational, patients may not have been interested in receiving even more information and may therefore have avoided logging in. In a future rollout of the MaMCVD intervention, the role of HCPs in referring patients to the web-based motivational and self-regulatory tools for patients will be emphasized more concretely, and sufficient patient numbers should be included to allow for investigating relationships between the use of web-based tools and PA outcomes. In addition, in future studies, the fidelity of MI sessions delivered to patients should be investigated as a potential moderator of subsequent engagement with web-based self-regulation tools and overall intervention effectiveness.

### Effects of the Web-Based Course for Health Care Professionals

Following completion of the web-based course, HCPs participating in this study self-reported considerable increases in self-efficacy for promoting PA in practice. This indicates that the web-based course helped HCPs become more confident that they could overcome the common barriers to promoting PA in practice. The intervention also led to moderate increases in planning and habits for PA promotion, meaning that PA promotion became somewhat more routine for HCPs after they completed the web-based course. As self-efficacy, planning, and habits have previously been shown to be strong predictors of other preventive clinical behaviors (eg, HCP behaviors in diabetes care) [77], it is reasonable to assume that HCPs who followed this web-based course might be more likely to promote PA to their patients outside the context of this study. HCPs reported no changes in their attitudes or intentions to promote PA, perhaps because of ceiling effects from the high baseline levels of these variables. Taken together, the effect sizes obtained for increases in self-efficacy and habit indicate that the HCP-facing MaMCVD intervention could help to improve PA promotion in practice. However, given the small sample size, potential bias of self-report measures, and lack of a control group in this study, these results should be interpreted with caution. Therefore, future studies may wish to investigate whether changes in self-efficacy, planning, and habit translate into objective changes in HCPs’ in-session PA promotion behaviors.

### Effects of the Interventions for Patients

Between the baseline and posttreatment time points, objectively assessed MVPA increased by nearly 10 minutes per day among patients with an adequate accelerometer wear time. Subjective measures of PA corroborated these results, with the total self-reported MVPA increasing by approximately 45 minutes per week after the intervention. These 3-month effects on objective and subjective levels of MVPA are similar in magnitude to those obtained from primary care–delivered PA promotion interventions in general [78]. However, it should be noted that the sample of participants in this study was already highly active at baseline, with >80 minutes of objectively assessed MVPA per day. Future testing of MaMCVD should try to overcome this self-selection bias and recruit a less active sample to maximize the potential impact on CVD risk.

In addition to increases in PA, participants reported beneficial changes in important motivational and volitional predictors of PA behavior. Intention for PA, the seminal predictor of PA in many behavioral theories, also increased from baseline to posttreatment. This represents an important outcome of the intervention as, according to the Rubicon model [79], solid intentions to perform a behavior are prerequisites for engaging in self-regulatory behaviors, such as goal setting and action planning. Theoretically, this fits well with the increased use of action planning for PA participants reported between baseline and posttreatment time points. Although self-reported action planning did increase, this was not borne out in the objective data on patients’ use of web-based self-regulatory tools, wherein only 11 participants set a goal or made an action plan. Therefore, self-reported increases in action planning more likely reflect individuals’ mental conceptualizations of future PA-related actions, as opposed to any specific written action plans.

Patients also reported increases in action control for PA, which entails a greater focus on efforts to increase PA behavior. This, too, is an important finding, as self-regulatory efforts to change behavior require increased attention to the target behavior to be effective [32]. A recent meta-analysis indicated that coupling these self-regulatory techniques with opportunities for patients to engage in supervised PA could increase these effects on intention [80], a possible add-on for future iterations of the MaMCVD intervention.

Although most intervention effects occurred in the intended direction, several did not. Introjected motivation and external motivation, controlled forms of motivation that may undermine long-term PA participation [81], both increased between baseline and posttreatment time points. Participants also reported less concern about developing CVD after treatment than they did at baseline, as well as a decrease in the extent to which they thought PA could prevent CVD incidence. Although the magnitudes of these potential adverse effects were small, any subsequent iterations of MaMCVD should seek to mitigate these effects. This could be done by emphasizing and supporting participants’ autonomy in their PA journeys by ensuring that participants engage with the educational content about the links between PA and CVD incidence and by clearly communicating CVD risk.

### Strengths and Limitations

This pilot study of the Movement as Medicine suite of behavior change interventions for HCPs and patients represents an important step in meeting the needs of HCPs tasked with promoting PA in primary care settings. The interventions were specifically designed to address HCPs’ barriers to PA promotion in practice and were designed in line with theory and evidence on how to increase motivation and PA among patients. In addition, the study used objective measurements of PA to overcome social desirability and other response biases in PA intervention studies [82].

Despite these strengths, some limitations should be considered. First, no clinical outcomes were assessed as part of this study, as we did not expect changes in these within the short 3-month study period. The possibilities of sampling bias (eg, more motivated participants enrolling in the study), ceiling effects, and response desirability bias on subjective outcome measures should also be considered as limitations of this study. Common biomedical measures associated with CVD risk, such as blood pressure, cholesterol, weight, and waist circumference, as well as objective measures where possible, should be included in future tests of this intervention. Second, the recruitment of primary care organizations fell below anticipated targets, which limits the ability to generalize patient retention rates across sites. This low uptake among primary care organizations also led to deviations from the published trial registration and a reduction in the scope of the study from an RCT to a nonrandomized pilot feasibility study. As a result, patient recruitment too fell below the initial sample size targets, meaning that we were unable to investigate the extent to which changes in the theoretical determinants of PA and patient engagement with the MaMCVD intervention contributed to levels of PA at the end of the intervention in this study. More financial resources and adoption to the NIHR portfolio would likely improve the uptake of the intervention and allow for testing of these predictive hypotheses. Finally, as this was a single-group pilot study, the reported effect sizes for changes in the variables under study should not be interpreted as causal or as estimates of the true effects. Rather, these effect size estimates should be used to calculate the sample size needed to demonstrate between-group efficacy in a larger RCT.

### Conclusions

The MaMCVD program sought to provide primary HCPs with new skills to promote PA during brief primary care consultations and, more broadly, to offer a follow-on PA treatment pathway for individuals with elevated CVD risk. The intervention improved self-efficacy for PA promotion among HCPs and may have had knock-on effects on their day-to-day practice. For patients participating in the study, MaMCVD offered web-based tools with which they could motivate themselves and self-regulate their efforts at PA behavior change. The intervention led to increases in the self-reported determinants of PA, including self-efficacy, intrinsic motivation, action planning, and action control, as well as increases in objectively assessed MVPA. Although these preliminary results indicate support for the program, the findings should be tempered, given the small sample size, absence of a control group, and use of self-report measures. In addition, feasibility problems around the uptake of the program by primary care organizations and national health research bodies need to be addressed before any broader rollout or testing of the program can take place. The results obtained here will be useful in improving these aspects of the MaMCVD program and can inform subsequent testing of the intervention in an RCT.

## Appendix

Multimedia Appendix 1 Supplementary materials, including screenshots of the websites for patients and health care providers, and the topic guide for interviews with health care providers.

## Abbreviations

BCT: behavior change technique
CPD: continuing professional development
CVD: cardiovascular disease
GP: general practitioner
HAPA: Health Action Process Approach
HCP: health care provider
MaMCVD: Movement as Medicine for CVD Prevention
MI: motivational interviewing
MVPA: moderate to vigorous physical activity
NHS: National Health Service
NIHR: National Institute for Health Research
PA: physical activity
PAR-Q: Physical Activity Readiness Questionnaire
RCT: randomized controlled trial
SDT: Self-Determination Theory
