# Supplementary material for: Movement as Medicine for Cardiovascular Disease Prevention: Pilot Feasibility Study of a Physical Activity Promotion Intervention for At-Risk Patients in Primary Care
Source: JMIR Cardio. 2022 Jun 29;6(1):e29035. doi: 10.2196/29035 (PMC9280491; doi:10.2196/29035)
Supplement: Multimedia Appendix 1 [file cardio_v6i1e29035_app1.pdf]

Supplementary Material

Contents	Page #
Screenshots of online interventions for HCPs	1-3
Screenshots of online interventions for Patients	4-8
Topic guide for qualitative interviews with HCPs	9

Screenshots of the MaMCVD interventions

Interventions for HCPs

Choice of modules

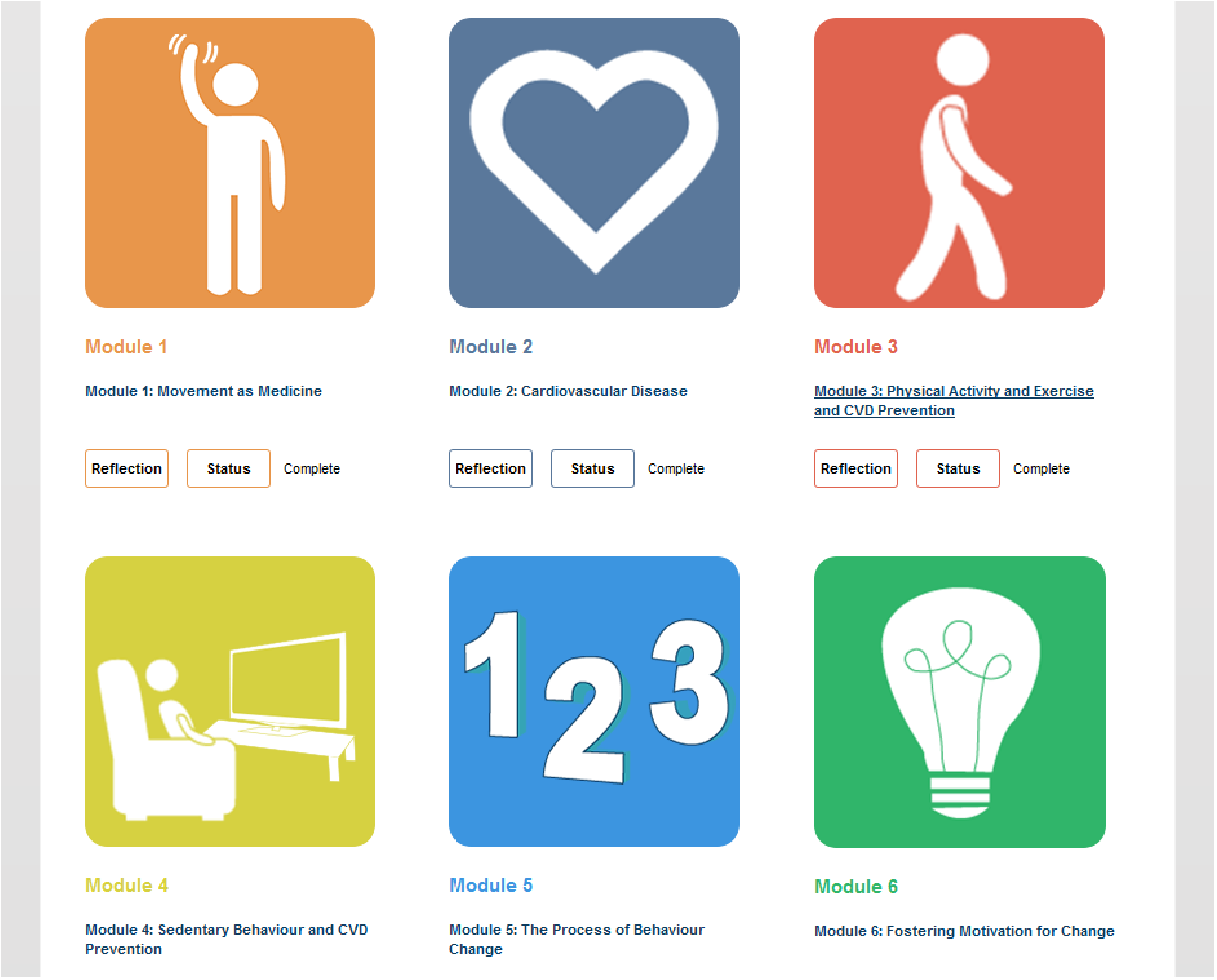

Interactive elements

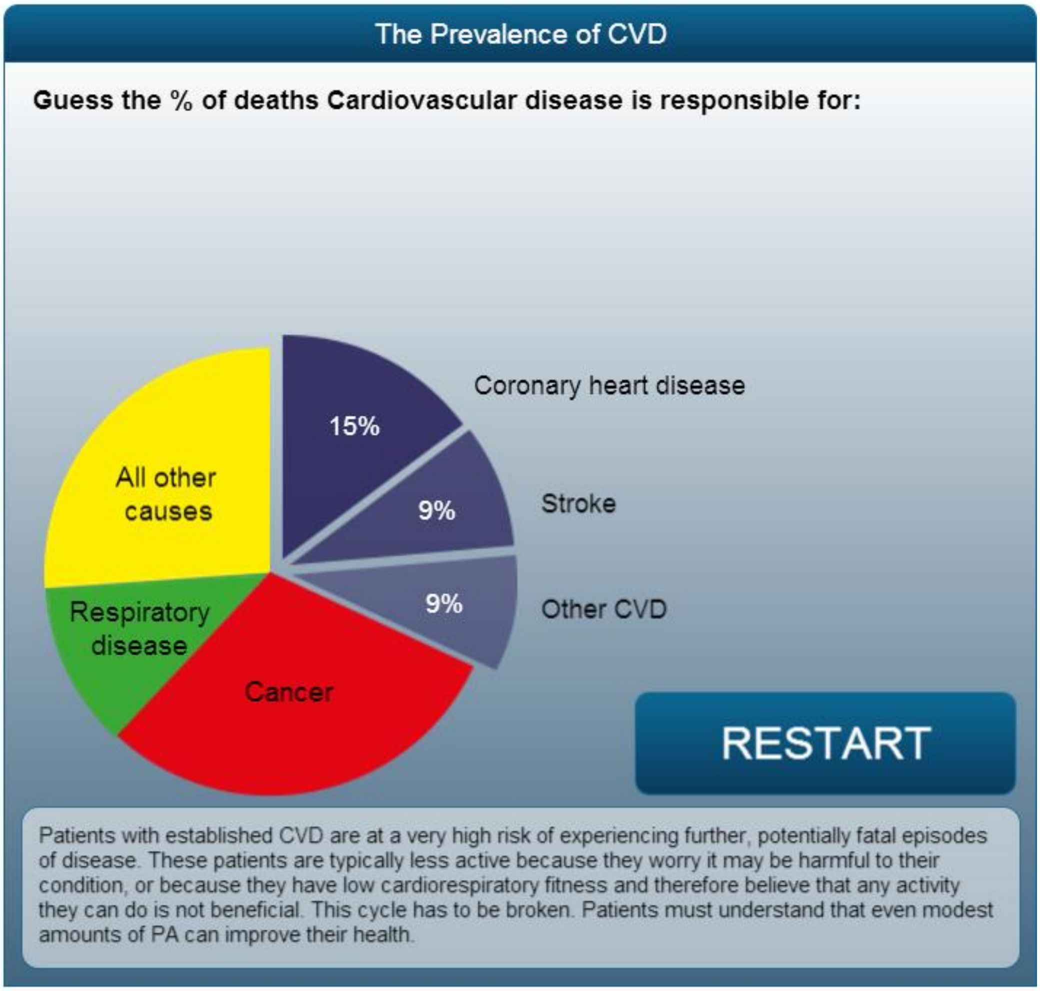

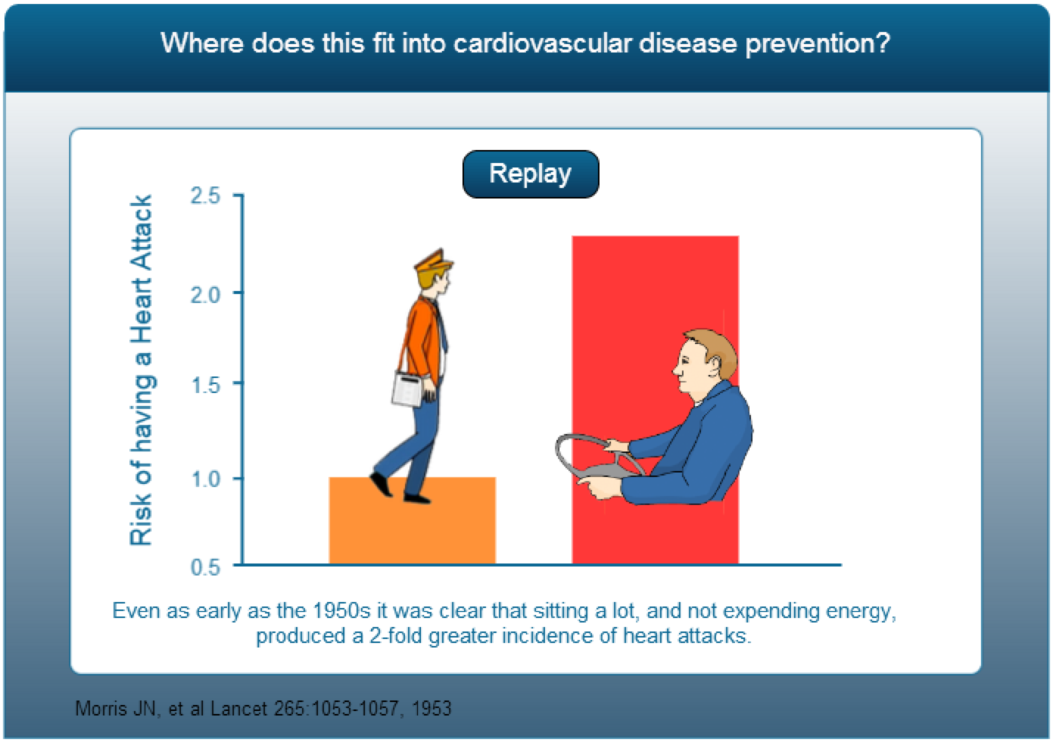

Types of change talk

Change talk includes statements in favor of change and against maintaining the status quo - hence the opposite of sustain talk. Change talk includes statements that expresses a **D**esire, **A**bility, **R**easons or **N**eed for change, as well as those that express a **C**ommitment to change and indications that someone has already **T**aken steps to change (**DARN-CaT**).

Use the buttons below to explore these various types of change talk.

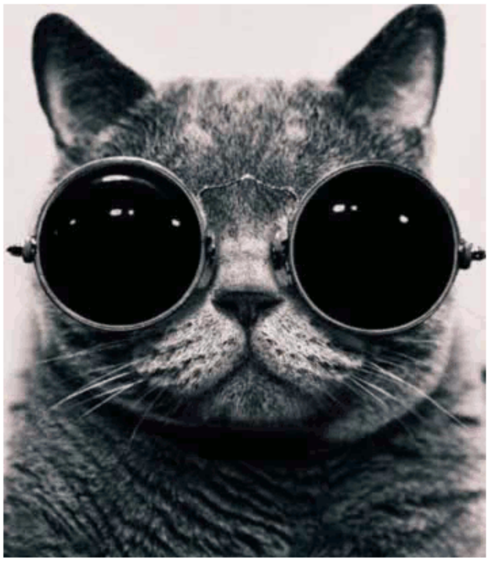

Desire | Ability | Reasons | Needs | Commitment | Taking steps

Ability

Change talk may also reflect a belief in capabilities for change.

This type of change talk is usually recognizable by inclusion of the words **can** or **could**, and sometimes by an individual's assessment of their own **ability**.

> I could probably do more to maintain/improve my health
>
> There are ways that I can be active, even if time is short
>
> I think I'd be able to do something like that, as long as I had support

10 Module 10: Self-regulation and Online Tools

Your Progress 1 2 3 4 5 6 7 8 9 10 11 12 13 14 15 16 17 18

Bridging the Gap

Over the last four modules you learned various techniques and strategies that you can apply in face-to-face behaviour change consultations to help patients form intentions to become more physically active (i.e. talk themselves into changing). While this is not always easy, intention formation is a necessary first step to creating any lasting improvements in health behaviours.

Once an intention is formed, an additional set of techniques are needed to help turn that intention into action, for as we discussed in module 5, research indicates that intention only leads to behaviour about a quarter of the time. This module will introduce these techniques, and walk you through a website that has been developed to help patients bridge their own intention-behaviour gaps.

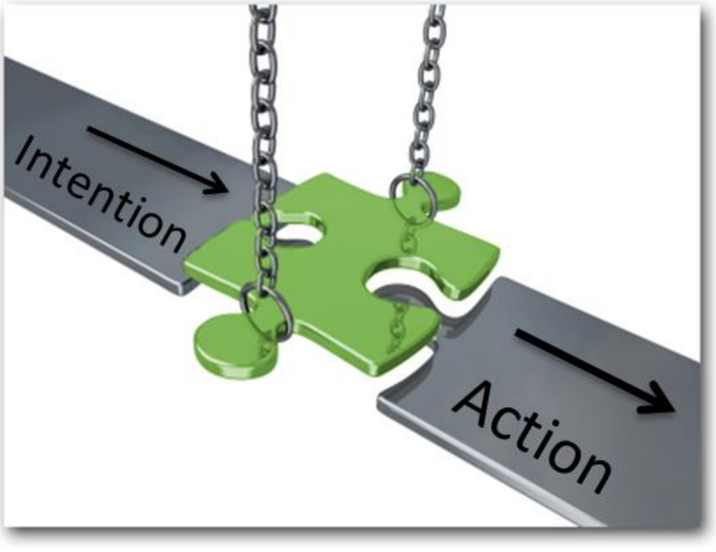

Interventions for Patients

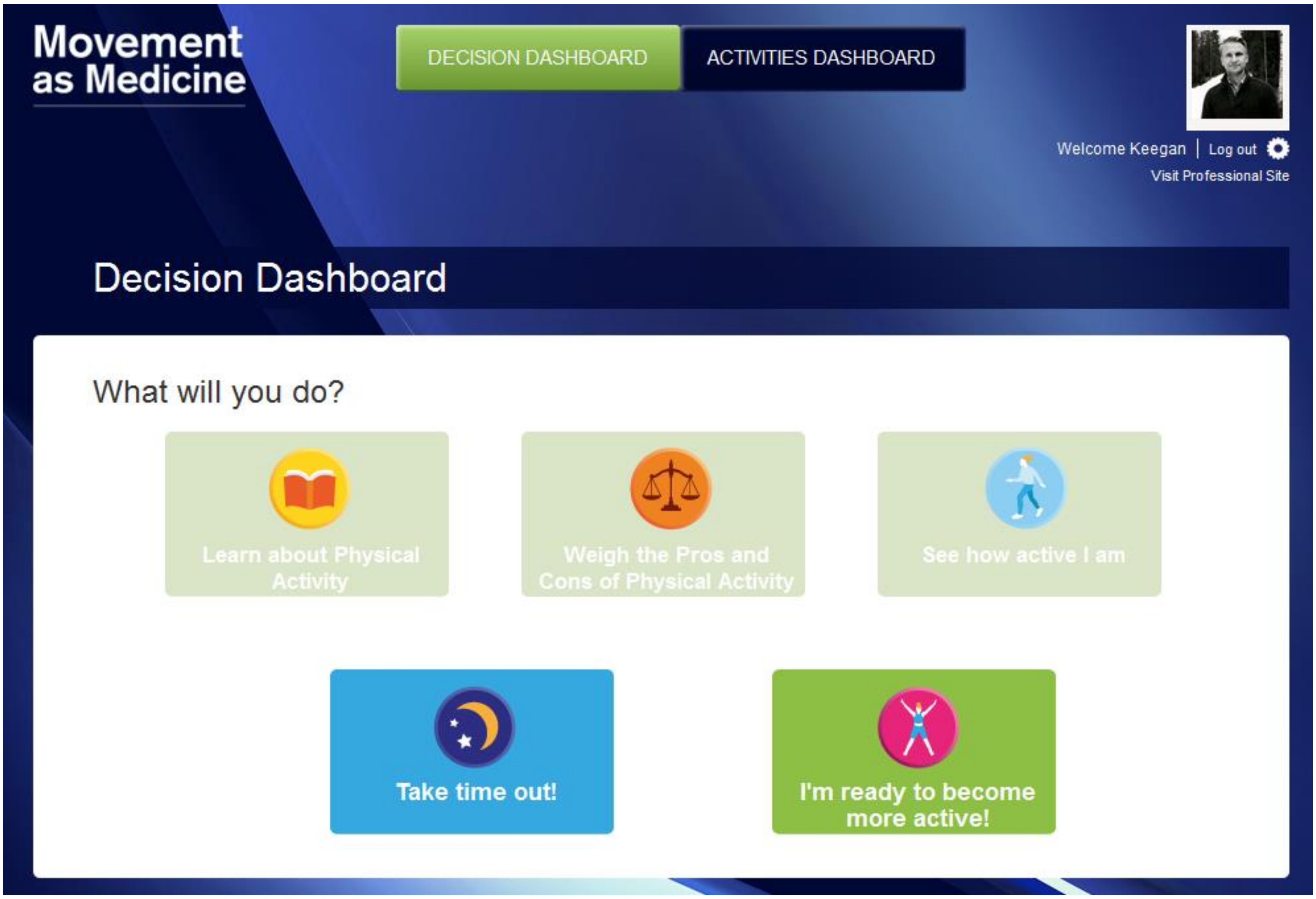

The Decision Dashboard contained the main motivational tools for patients and was intended to help them develop and formulate their intentions for wanting to become physically active..

Clicking on 'Learn about Physical Activity' would take participants to a menu of brief e-learning modules providing info about the benefits of physical activity and tips on getting started. A fuller description is available in the article. See page 5 for a screenshot.

'Weigh the Pros and Cons' took participants to the decisional balance tool. See page 5 for a screenshot.

The 'See how active I am' initiated a one-week self-monitoring period, wherein individuals would track their physical activity. At the end of this, they would receive a report on how their current levels compare to recommended levels of PA.

'Take time out' allowed participants to step away from their physical activity journey for an amount of time that they themselves chose. The system would then send them a notification via email or SMS at the end of this specified period.

Clicking 'I'm ready to become more active' would take users to a page where they could specify their own reasons for wanting to become more active. If they could not think of any specific reason, then they were prompted to complete the Motivation Assessment tool shown on page 6.

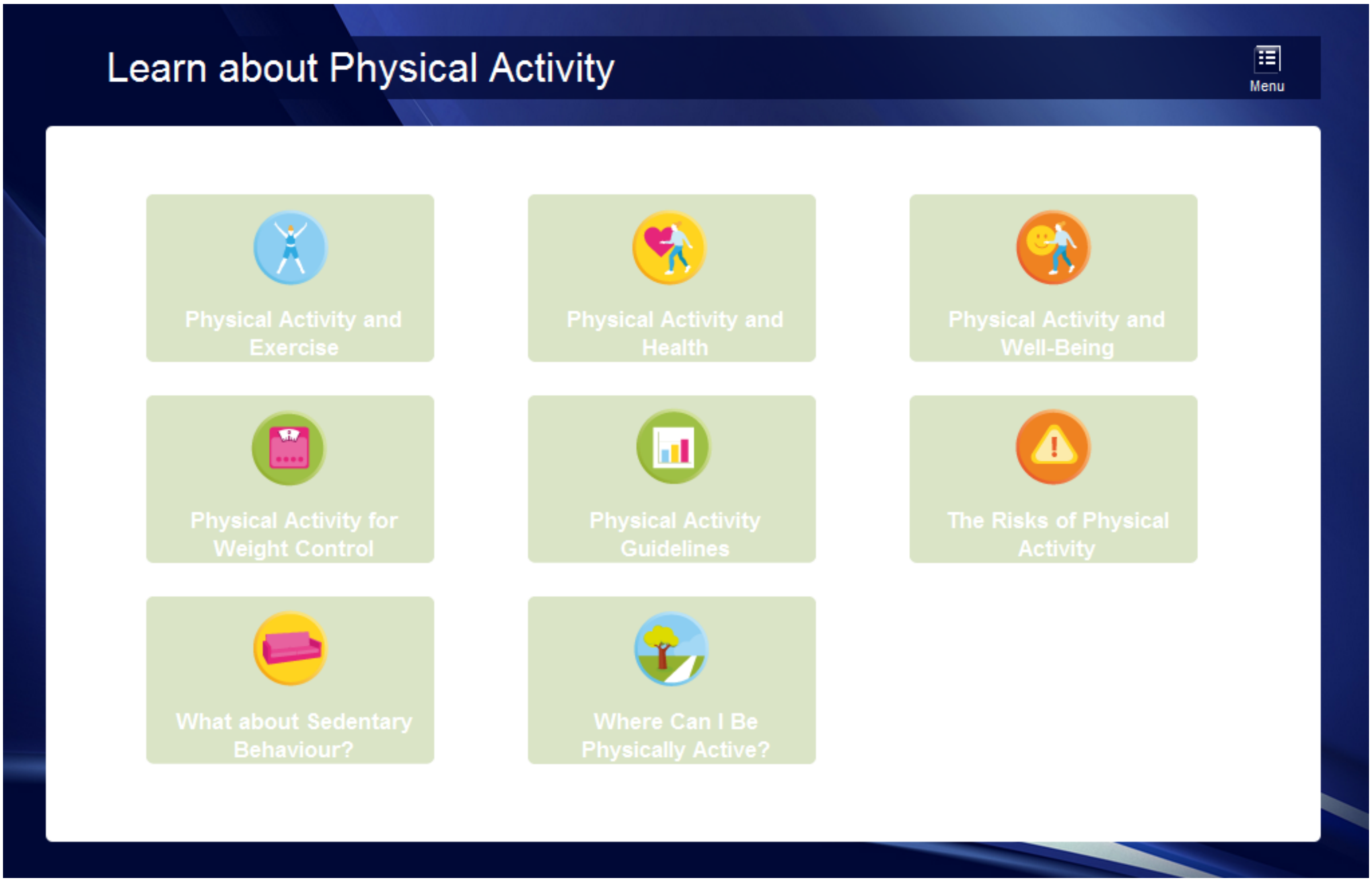
Learn about Physical Activity
Menu
Physical Activity and Exercise
Physical Activity and Health
Physical Activity and Well-Being
Physical Activity for Weight Control
Physical Activity Guidelines
The Risks of Physical Activity
What about Sedentary Behaviour?
Where Can I Be Physically Active?

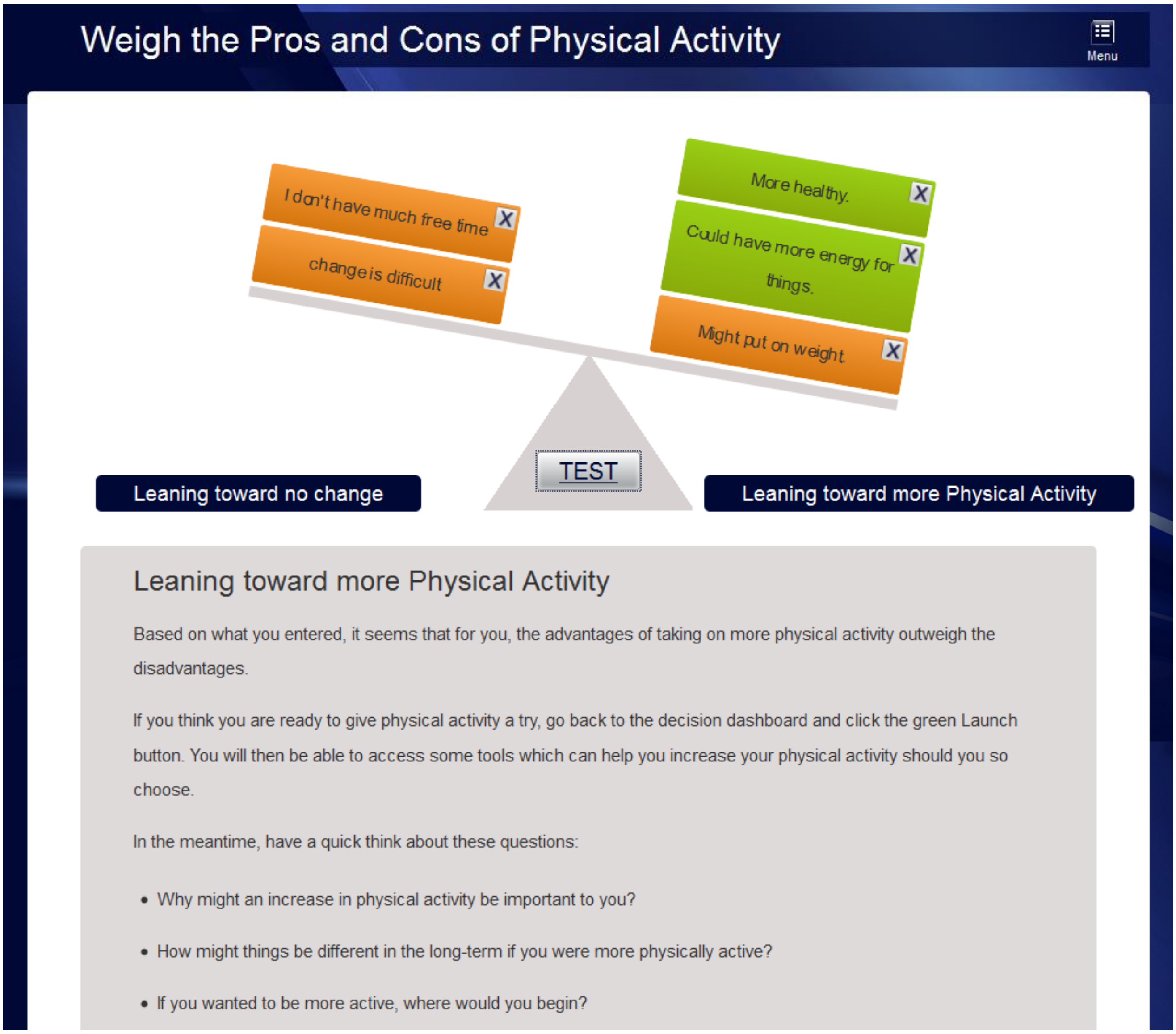
Weigh the Pros and Cons of Physical Activity
Menu
I don't have much free time
change is difficult
More healthy.
Could have more energy for things.
Might put on weight.
TEST
Leaning toward no change
Leaning toward more Physical Activity
Leaning toward more Physical Activity
Based on what you entered, it seems that for you, the advantages of taking on more physical activity outweigh the disadvantages.
If you think you are ready to give physical activity a try, go back to the decision dashboard and click the green Launch button. You will then be able to access some tools which can help you increase your physical activity should you so choose.
In the meantime, have a quick think about these questions:
• Why might an increase in physical activity be important to you?
• How might things be different in the long-term if you were more physically active?
• If you wanted to be more active, where would you begin?

Motivation Assessment Tool

What might you personally stand to gain from becoming (more) physically active? Read each of the statements below, and indicate the extent to which you agree or disagree. At the end, click on submit to find out how physical activity might help you to get more of what you want out of life.

I would like to look more physically attractive

Do not agree at all — Agree fully

I would like to gain recognition for my accomplishments

Do not agree at all — Agree fully

I would like to maintain good health

Do not agree at all — Agree fully

I would like to feel more healthy

Do not agree at all — Agree fully

I would like to prevent health problems/complications

Do not agree at all — Agree fully

I am concerned about developing heart disease

Do not agree at all — Agree fully

Motivation Assessment Tool

Based on your responses, the following reasons might motivate you to become (or remain) physically active:

- **Curiosity** (90% match).
  View details...
- **Affiliation** (90% match).
  View details...
- **Competition** (80% match).
  View details...
- **Weight Management** (70% match).
  View details...
- **Positive Health** (70% match).
  View details...

Curiosity

Try new things!

Curiosity may have killed the cat, but for you, it's a driving force! Physical activity is a great way to try new things, particularly if you have not been very active in the past.

If you want to try new things, some of the physical activities below might be a good place to start. Or, if physical activity is new to you, then maybe try starting with more traditional activities like walking or cycling.

- Surfing
- Tai Chi or Capoeira
- Disc golf
- Ballroom dancing
- You might consider one of the following for your motivation: I want to become more physically active so that I can...

Activities Dashboard

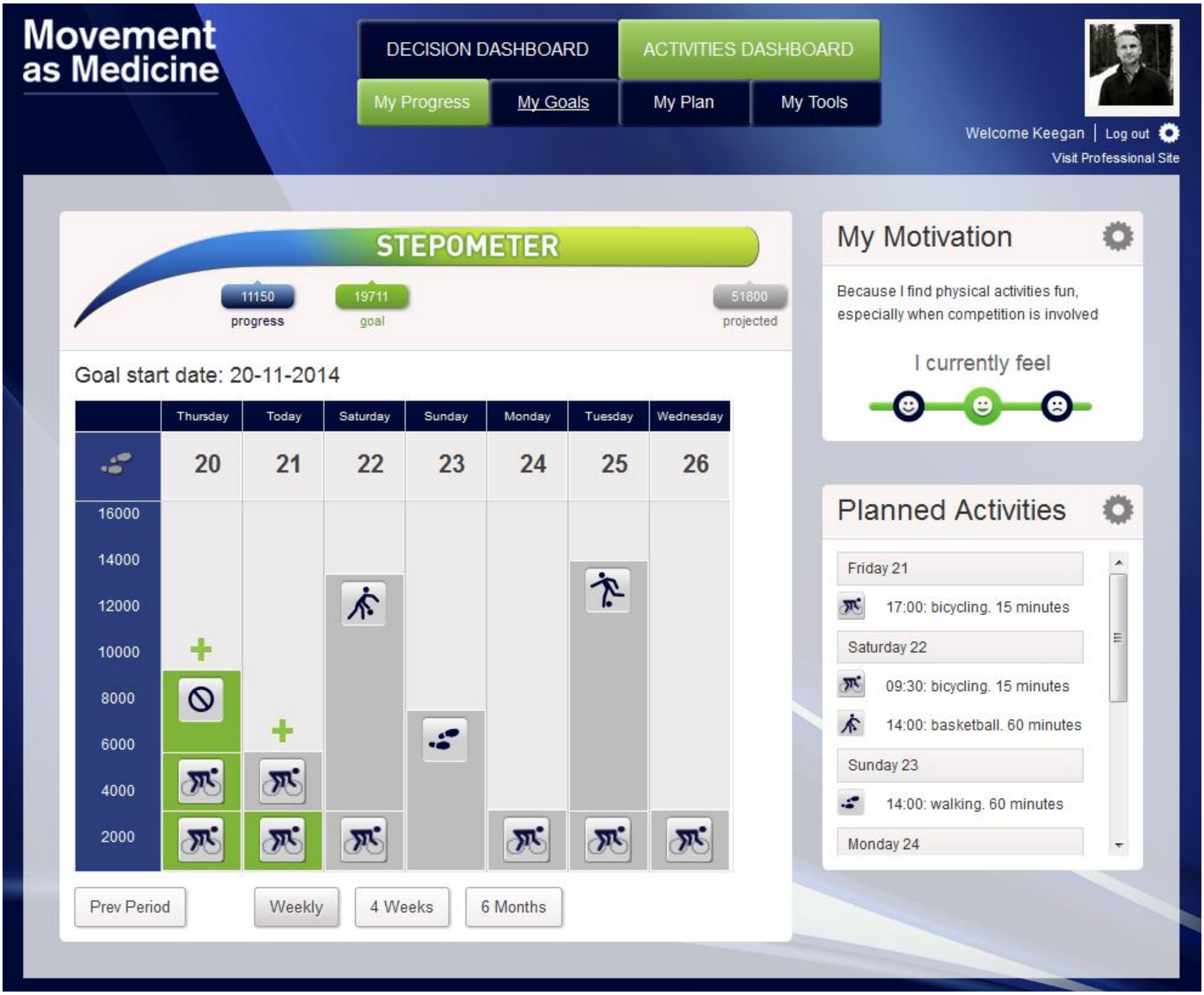

The Activities Dashboard contained the main self-regulation tools for patients.

The Stepometer at the top shows completed steps in blue (‘progress’), goal steps in green (‘goal’), and planned plus completed steps in grey (‘projected’).

The main calendar view shows blocks for completed activities in green, and planned activities in grey. The sizes of block are proportional to the number of steps each activity represents. Participants can log steps or activities by clicking the green plus signs.

Participants’ stated motivations for wanting to become more physically active are shown at the top right in the My Motivation section. Clicking on the settings gear would take participants to a different page where they could edit this information. Participants could also self-monitor their mood in this area.

Upcoming planned activities are shown in the Planned Activities area on the bottom right. Clicking on the settings gear would take participants to a different page where they could add activities to their plan.

Problem Solving Tool

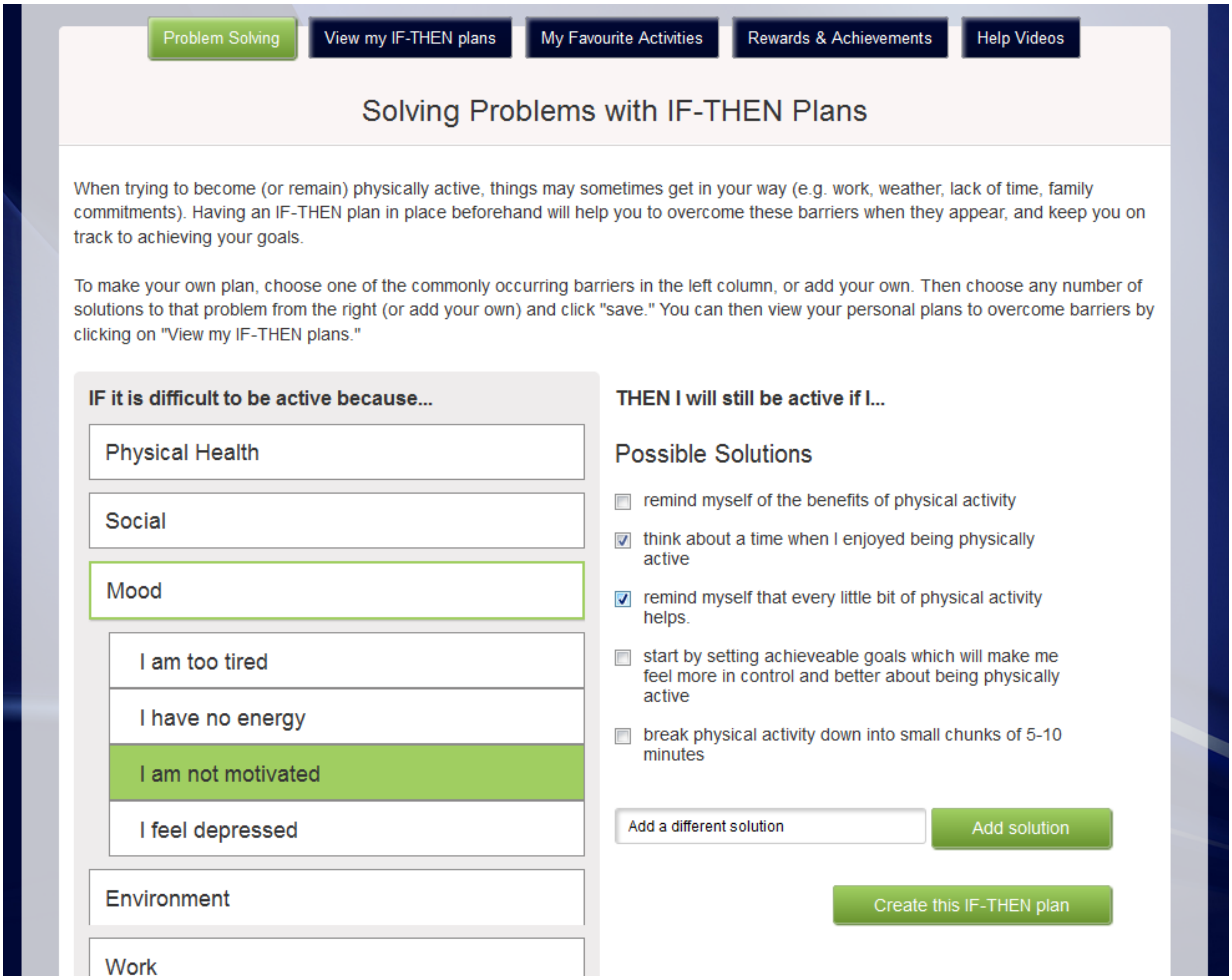

The Problem Solving Tool was found on the ‘My Tools’ section of the Decision Dashboard. It allowed people to choose situations in which they found it to be physically active (i.e. barriers) in the left panel and to link these to possible ways to overcome these barriers (i.e. solutions) in the right panel. People could add their own barriers and solutions if they could not find any suitable ones in the menus.

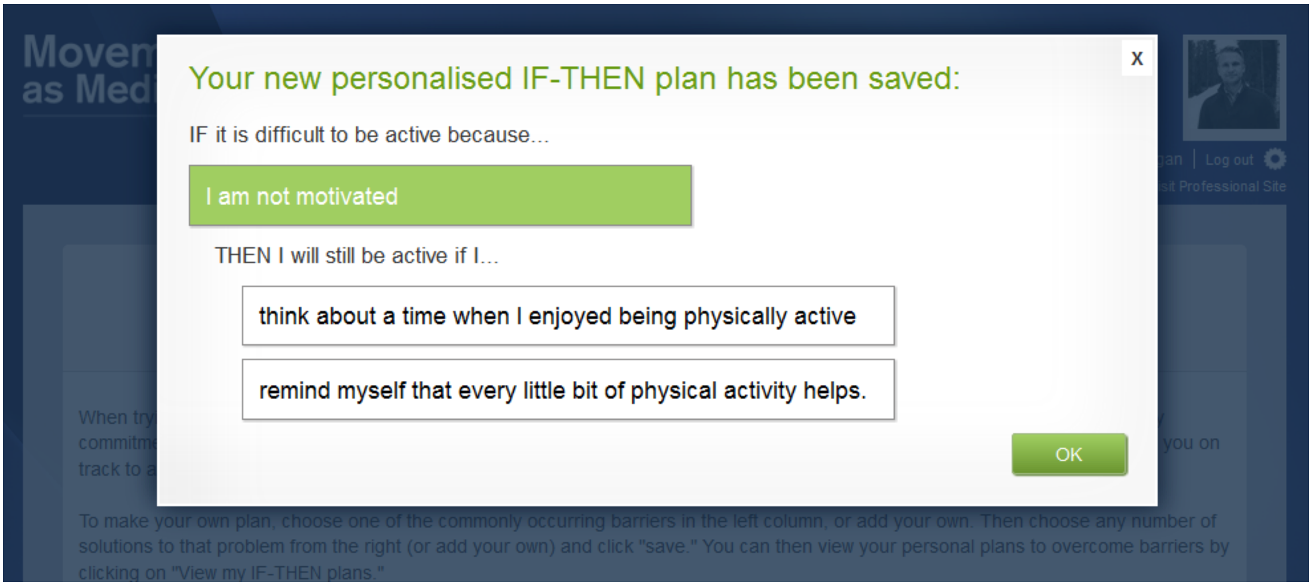

After selecting their barriers and solutions, the completed IF-THEN plans were presented to participants and stored in the ‘View my IF-THEN plans’ section.

Interview Schedules for Participating Healthcare Professionals

Interview 1: Experiences with Online Educational Material and Expectations for Delivery
When: Before delivery of first Movement as Medicine session with patient
Duration: 10-20 minutes

Introduction: *You have recently completed the Movement as Medicine online course, and are about to have your first consultation with a Movement as Medicine patient. In this interview we would like to ask you some questions about your experiences with the online course, and about your expectations for the upcoming face-to-face consultations. Would that be alright? Also, would it be alright if I record this interview? Your responses will be kept confidential, and used only for research purposes and to refine the Movement as Medicine program.*

<u>Topic 1: The online course</u>

- What are your thoughts on the website/course materials in general?
    - Is it: Appealing? Comprehensible? Credible? Fun? Modern? Professional?
    - How could the factors above be improved?
- How clear were the objectives of the course?
- What were your expectations going in?
- What were your main take home points from the course?
    - To what extent are these applicable in other domains of your practice outside of promoting regular physical activity?
- How applicable is the content of the course to your day-to-day work in primary care?
- To what extent did the course make you more confident of your ability to promote physical activity?
- Did you miss anything in the course?
- Was any of the content redundant or incorrect? How could the course be improved?
- Did you access the course via a mobile device or tablet?
- Was the amount of CPD credit appropriate for the course?

<u>Topic 2: Expectations for the face-to-face consultations</u>

- What are your expectations for the face-to-face sessions?
- Which key skills from the course do you plan to apply?
- What difficulties do you think you might have in applying the skills you learned?
- How confident are you that you can:
    - Create patient change talk?
    - Recognize patient change talk?
    - Respond to patient change talk?
- How do you think the sessions will differ from your standard consultations with patients?

Interview 2: Experiences with face-to-face consultations

When: After delivery of Movement as Medicine session to first 2 patients
Duration: 5-10 minutes
<u>Topic 1: Experiences with first face-to-face consultation</u>

- How did you experience this first session?
- What went well? What went less well?
- How do you think the patient experienced it?
- What impact did it have for them?
- What will you change/avoid doing in the next session?
- How well did the course prepare you for the session?
